# Chronic Cigarette Smoking: Implications for Neurocognition and Brain Neurobiology

**DOI:** 10.3390/ijerph7103760

**Published:** 2010-10-21

**Authors:** Timothy C. Durazzo, Dieter J. Meyerhoff, Sara Jo Nixon

**Affiliations:** 1 Department of Radiology and Biomedical Imaging, University of California, 505 Parnassus Avenue, M-391, San Francisco, CA 94143, USA; 2 Center for Imaging of Neurodegenerative Diseases, San Francisco VA Medical Center, 4150 Clement St., San Francisco, CA 94121, USA; E-Mail: dieter.meyerhoff@ucsf.edu (D.J.M.); 3 Department of Psychiatry, University of Florida, P.O. Box 100256, Gainesville, FL 32611, USA; E-Mail: sjnixon@ufl.edu (S.J.N); 4 Department of Psychology, University of Florida, P.O. Box 112250, Gainesville, FL 32611, USA

**Keywords:** chronic cigarette smoking, neurocognition, neurobiology, neuroimaging, genetics

## Abstract

Compared to the substantial volume of research on the general health consequences associated with chronic smoking, little research has been specifically devoted to the investigation of its effects on human neurobiology and neurocognition. This review summarizes the peer-reviewed literature on the neurocognitive and neurobiological implications of chronic cigarette smoking in cohorts that were not seeking treatment for substance use or psychiatric disorders. Studies that specifically assessed the neurocognitive or neurobiological (with emphasis on computed tomography and magnetic resonance-based neuroimaging studies) consequences of *chronic* smoking are highlighted. Chronic cigarette smoking appears to be associated with deficiencies in executive functions, cognitive flexibility, general intellectual abilities, learning and/or memory processing speed, and working memory. Chronic smoking is related to global brain atrophy and to structural and biochemical abnormalities in anterior frontal regions, subcortical nuclei and commissural white matter. Chronic smoking may also be associated with an increased risk for various forms of neurodegenerative diseases. The existing literature is limited by inconsistent accounting for potentially confounding biomedical and psychiatric conditions, focus on cross-sectional studies with middle aged and older adults and the absence of studies concurrently assessing neurocognitive, neurobiological and genetic factors in the same cohort. Consequently, the mechanisms promoting the neurocognitive and neurobiological abnormalities reported in chronic smokers are unclear. Longitudinal studies are needed to determine if the smoking-related neurobiological and neurocognitive abnormalities increase over time and/or show recovery with sustained smoking cessation.

## 1. Introduction

Approximately 2 billion people worldwide use tobacco products, mostly in the form of cigarettes, with tobacco smoking-related diseases resulting in 4 million deaths per year [1]. Among the approximately 64.5 million active smokers in the USA, smoking-related disease results in approximately 440,000 preventable annual deaths [2]. The enormous healthcare expenditures and mortality associated with chronic cigarette smoking results in an estimated $92 billion annual productivity loss in the US. Internationally, the greatest smoking related mortality is increasingly apparent among economically disadvantaged groups, which, in the US includes a disproportionate number of ethnic minorities and those with psychiatric and substance use disorders [3,4]. An extensive body of research thoroughly describes the deleterious effects of chronic cigarette smoking on human cardiac and pulmonary function, peripheral vascular systems as well as its carcinogenic properties [5–8]. Recent research indicates chronic cigarette smoking is associated with increased risk for numerous biomedical conditions that may directly or indirectly compromise brain neurobiology and neurocognition [9–12]. However, compared to the substantial volume of research on the cardiovascular, pulmonary and cancer-related health consequences associated with chronic smoking, surprisingly little research has been specifically devoted to the investigation of its effects on human neurocognition and brain neurobiology.

This review summarizes the peer-reviewed literature on the neurocognitive and neurobiological repercussions of *chronic* cigarette smoking in cohorts and population-based samples that were not specifically seeking treatment for substance use or psychiatric disorders. Prospective or retrospective studies that expressly assessed the neurocognitive or neurobiological consequences of chronic smoking are targeted. Research employing proton magnetic resonance-based studies of brain morphology and metabolites that specifically evaluated the neurobiological consequences of chronic smoking are emphasized. In this review, non-smoking control groups are referred to as NSC and individuals comprising these groups generally were indicated to be never smokers or consumed less than 100 cigarettes over lifetime. NSC were equivalent in age to smoking cohorts unless otherwise specified. The research reviewed was generally conducted with individuals in one of three age ranges: 18–30, 40–59 and 60–90. Individuals 18–30 years of age are referred to as “young adults”, 40–59 as “middle-aged adults” and 60–90 years of age as “older adults”. In studies where the participants do not conform to the above defined age groups, specific age ranges are provided. For reviews on the effects of chronic smoking on brain neurobiology and function in alcohol and substance use disorders see [13–15]. Please refer to [16–20] for thorough reviews on the *acute* effects of nicotine administration and nicotine withdrawal on brain neurobiology and neurocognition (although not the focus of this review, these topics are briefly addressed in Section 4). For inclusive reviews on functional MRI and nuclear imaging findings in chronic smokers see [21,22].

## 2. Neurocognitive Consequences of Chronic Cigarette Smoking (see Table 1)

The vast majority of research investigating the neurocognitive consequences of chronic cigarette smoking is cross-sectional in design and focused primarily on middle-aged and older adults. In the sole study of adolescents, daily smokers (mean age = 17 ± 1) showed deficits in accuracy of working memory relative to NSC, with individuals who began smoking at a younger age demonstrating greater impairment than those who began smoking at a later age [23]. In the few studies with young adults, smokers were inferior to NSC on measures of sustained attention and impulse control [24], auditory-verbal memory, oral arithmetic, and receptive and expressive vocabulary [25], information processing speed [26] and general intelligence [27]. On an experimental behavioral measure of risk-taking (Balloon Analogue Risk Task [28]), young adults smokers demonstrated higher levels of risk-taking [29]. In cross-sectional studies specifically comparing NSC to middle-aged and/or older adult smokers, poorer performance in smokers was reported for auditory-verbal learning and/or memory [30–34], working memory [26,35,36], executive functions [33,37,38], general intellectual abilities [39], visual search speed [40], processing speed and cognitive flexibility [30–32,38,41,42] and global cognitive function (e.g., brief mental status examinations such as the MMSE) [41]. In a middle-aged cohort of combined current and former smokers, any history of smoking was associated with increased risk for abnormal auditory-verbal memory [43]. Some studies observed the performance of former smokers fell between that of current smokers and NSC in young [25], middle-aged and older adults [31,35,39]. Other studies found no differences between former smokers and NSC [25,30]. The inconsistencies among these studies may be related to the substantial variety of measures used across studies to evaluate the domain of functioning in question as well as inconsistency in the magnitude of neurocognitive dysfunction in the smoking study cohort.

In cross-sectional population-based studies with community-dwelling older adults, where smoking status (*i.e.*, current smoker, past smoker, never smoker) was used as a prospective or retrospective predictor, current smoking [44–46] and any history of smoking [47] were associated with poorer performances on measures of global cognitive function. Any previous smoking history (with variable lengths of smoking cessation) was associated with poorer cognitive flexibility [45], and impaired general cognitive function [44] or, conversely, decreased risk of global cognitive impairment [46]. Chronic smoking in older adults has also been associated with a diminished ability to execute some activities of daily living [48] and compromised postural stability [49].

Longitudinal research with non-demented, population-based samples found that current cigarette smokers demonstrated an abnormal rate of decline on indices of reasoning [43] and auditory-verbal memory [40] in middle aged adults, and abnormal decline on measures of global cognitive function [47,50–53] and auditory-verbal memory in older adults [54].

Sex effects in neurocognition among smokers have also been addressed in some studies. Edelstein *et al.* [34] found no differences between older adult male smokers and male NSC on measures of global cognitive functioning, set-shifting, semantic fluency and auditory-verbal and visuospatial learning and memory, while older adult female smokers demonstrated poorer global cognitive functioning and auditory-verbal memory than female NSC. Jacobsen and colleagues [23] reported that male adolescent smokers performed more poorly than did female smokers on measures of selective and divided attention. Similarly, Razani and colleagues [37] observed that sex was a significant predictor of non-verbal abstraction in currently smoking older adults, however, no interactions were reported among sex, smoking status or smoking severity. Other studies, however, have found no sex effects on neurocognition in middle aged and older adults [32,33,39].

The level and chronicity of smoking, as reflected in the number of cigarettes smoked per day, duration of smoking over lifetime, and/or dose-duration (*i.e.*, pack-years) were inversely related to various domains of neurocognition in adults across a wide age range [25,30,32,45,52,55,56]. Several reports indicate chronic smoking is associated with increased risk for various forms of dementia, in particular Alzheimer’s Disease and vascular dementia [57–61]. This risk may be modulated through the apolipoprotein E ɛ4 (ApoE4) genotype, a known genetic risk for the development of Alzheimer’s Disease [52,54]. Interestingly, some studies have reported that risk for development of Alzheimer’s Disease was greater in smokers who were *not* ApoE4 carriers [52,54,58,59].

Several studies, smoking status (*i.e.*, smoker or non-smoker) or measures of smoking consumption (e.g., pack years), showed weak or no relationships to specific neurocognitive functions (e.g., measures of learning and memory, mental arithmetic, verbal fluency, processing speed), global neurocognitive function (e.g., MMSE) and neurocognitive decline in young and middle aged adults [62,63] and in large community-based samples consisting of middle-aged and older adults [64–70].

## 3. Neurobiological Consequences of Chronic Cigarette Smoking (See Table 2)

The specific neurobiological factors underlying the reported smoking-related cognitive deficits are not established. However, there are a few of computed tomography (CT) and magnetic resonance (MR)-based studies that suggest the reported neurocognitive deficiencies in smokers may be, in part, mediated by abnormalities in brain morphology, perfusion and/or neurochemistry. The majority of these studies are cross-sectional in design.

### 3.1. Brain Morphology

Computed tomography (CT) studies with cohorts ranging from middle-aged to older adults report that chronic smoking is associated with an abnormal increase of global brain atrophy with advancing age [74–77]. These CT studies assessed whole brain volumes and did not report major anatomical subdivisions (e.g., frontal gray matter/white matter). An early MRI study with older adults examined global brain atrophy over a 5-year-interval and found higher pack years was related to increased ventricular volume in men and was associated with increased sulcal volume in women, after controlling for age and vascular risk factors [78]. More recent MRI studies have employed voxel-based morphological measures to assess the regional brain volumes and densities of the cortical gray matter (GM). Smokers aged 39.5 ± 10.3 years evidenced smaller volumes and lower tissue densities than did NSC in bilateral anterior frontal lobe regions; smokers also had smaller volume of the left dorsal cingulate cortex and lower GM density in the cerebellum. Anterior frontal cortex density was inversely related to pack-years [79]. Smokers aged 30.8 ± 7.5 years demonstrated widespread GM volume and density reductions relative to NSC [80], particularly in the bilateral frontal lobes, cingulate gyrus and insula. Non-demented older adult smokers (75.0 ± 3.4 years of age) exhibited reduced GM density in right precuneus, left posterior cingulate gyrus, right thalamus and bilateral precentral and middle frontal gyri compared to NSC [81].

A MR-based study employed diffusion tensor imaging (DTI) and voxel based morphometry to assess microstructural integrity and morphology, respectively, of the corpus callosum in middle aged chronic smokers [82]. Contrary to expectations, smokers demonstrated higher fractional anisotropy (FA; higher FA values are considered to reflect greater microstructural integrity [83,84]) in the body and total corpus callosum than did NSC and no volume differences were observed between smokers and NSC in the corpus callosum. However, smokers with high levels of nicotine dependence (as reflected by scores on the Fagerstrom Test for Nicotine Dependence) had significantly lower FA values than both smokers with low levels of nicotine dependence and NSC. It is widely recognized from population-based studies, with middle-aged and older adults, that chronic smoking is associated with increased incidence of regional white matter (WM) signal hyperintensities on standard MR imaging (e.g., T2-weighted and FLAIR) [85–89]. WM hyperintensities are associated with decreased cerebral blood perfusion [90,91] and neurocognitive dysfunction [92,93]. Overall, the degree of smoking-related morphological changes observed appears to be contingent on the method and brain region under consideration.

### 3.2. Brain Biochemistry

A single volume proton (^1^H) MR spectroscopy study with chronic smokers (36 ± 11 years of age) observed lower N-acetylaspartate (NAA) concentration (surrogate marker of neuronal integrity [94,95]), in the left hippocampus relative to NSC. No group differences were observed for NAA in the anterior cingulate cortex (ACC), but choline-containing compound (Cho) levels (a marker of cell membrane turnover/synthesis [94,96]) were positively related to greater pack years in this region [97]. A single voxel ^1^H spectroscopy study of glutamate levels in the left hippocampus and ACC observed no differences among current smokers (35 ± 10 years of age), former smokers (42 ± 10 years of age) abstinent for 17 ± 3 years and NSC (33 ± 10 years of age) [98]. In the sole ^1^H spectroscopy study investigating gamma aminobutyric acid levels (GABA; neuromodulator involved in the development and maintenance of substance use disorders [99–101]) in chronic smokers, cortical GABA concentrations were lower in female smokers (and modulated by menstrual cycle phase), but GABA levels were not different between male smokers and NSC [102].

### 3.3. Brain Perfusion

The vast majority of neuroimaging research on brain perfusion has investigated the effects of acute nicotine exposure, rather than the consequences of chronic cigarette smoking [18]. The few published reports specifically investigating chronic smokers indicate globally decreased brain perfusion relative to NSC, as measured by CT ^133^Xe inhalation [103,104] in older adults and single proton emission computed tomography (SPECT) [105] in adults aged 35.5 ± 8.4 years; perfusion was inversely related to cigarette pack-years [105]. In a Xe-CT-based longitudinal study with community-dwelling older adults, decreases in global cerebral perfusion were independently associated with chronic smoking controlling for other vascular risk factors [106,107].

## 4. Neurocognitive and Neurobiological Effects of Acute Nicotine Exposure and Withdrawal

When investigating chronic cigarette smoking-induced neurobiological and neurocognitive dysfunction alone, or in conjunction with AUD and other conditions, it is important to distinguish the effects of acute nicotine ingestion and withdrawal from the potential consequences of chronic exposure to the multitude of noxious compounds contained in cigarette smoke. While not the focus of this review, the general findings and implications are discussed regarding the effects of acute nicotine on neurocognition and brain neurobiology, as measured with functional neuroimaging methods [*i.e.*, functional MRI (fMRI), positron emission tomography (PET), single positron emission tomography (SPECT)].

### 4.1. Acute Nicotine Consumption, Nicotine Withdrawal and Neurocognition

Acute nicotine administration has been found to transiently improve some areas of neurocognition in NSC and individuals with attention deficit hyperactivity disorder and schizophrenia-spectrum disorders, most substantially on measures of sustained attention and working memory [17,19,108]. Acute nicotine administration in nicotine deprived smokers is associated with improved cognitive task performance [109,110], whereas several studies report decrements in neurocognitive performance with nicotine administration to NSC (see [19] for review). A recent meta-analysis conducted by Heishman and colleagues [111] suggests that acute smoking or nicotine consumption, independent of withdrawal effects, is associated with enhanced function in the following domains of function: fine motor skills, alerting attention accuracy and response time, orienting attention reaction time, short-term episodic memory accuracy and working memory reaction time (but not accuracy). In non-clinical chronic smokers, the adverse effects of nicotine withdrawal are not typically apparent on neurocognitive function until 8–12 hours after last nicotine dose [17,19,109,112]. Protracted duration from last cigarette smoked/nicotine administration to onset of withdrawal mediated disturbances in neurocognition is likely attributable to the maintenance of relatively high levels of plasma nicotine during waking hours due to repeated dosing of nicotine (via cigarettes) [113].

### 4.2. Acute Nicotine Consumption, Nicotine Withdrawal and Neurobiological Function

Several functional neuroimaging (PET, SPECT, fMRI) studies in active chronic smokers (see [21,22] for review) and a few functional MRI studies addressed the acute effects of nicotine administration on brain activity during task activation in healthy non-smokers [17,18,20]. The effects of acute cigarette smoking on functional neuroimaging modalities in non-smokers have not been investigated [18,20]. In chronic smokers, functional neuroimaging studies investigating responses to acute smoking or nicotine administration have shown are that acute nicotine administration is associated with decreased global cerebral blood flow, increased activity in the dorsolateral, inferior and mesial frontal and orbitofrontal regions, thalamus and visual processing regions (see [21,22]). In chronic smokers deprived of tobacco for more than 2 hours, acute cigarette smoking elicits different patterns of relative perfusion responses, with increases of the order of 6–8% in the anterior frontal and cingulate cortices as well as decreases in cerebellum and occipital lobes that were associated with plasma nicotine levels [18,114,115]. Some studies report a 7–10% decrease in global glucose utilization following acute nicotine administration in chronic smokers deprived of nicotine for 8 hours or more [116,117]. Depending on the nature of the task, results suggest acute nicotine administration in smokers and non-smokers is associated with increased regional blood flow/brain activity and improves task performance or decreases blood flow/oxygenation level-dependent activity and task performance [18,20]. As discussed by Sharma and Brody [22], the reported regionally specific findings may be influenced by whether or not activity was standardized to whole brain blood flow.

Overall, the effects of acute nicotine administration on neurocognition and functional imaging measures appear to depend on duration of nicotine deprivation, the brain region studied, resting *versus* activation conditions, and the neurocognitive domain investigated [18].

## 5. Potential Biological Mechanisms Contributing to Chronic Cigarette Smoking-Induced Neurocognitive and Neurobiological Dysfunction

Nicotine is one of more than 4000 compounds composing the particulate and gas phases of cigarette smoke [5,8,118]. In addition to nicotine, scores of these compounds are bioactive and may affect tissue locally in the oral cavity, the upper and lower respiratory systems, and distally via the systemic circulation. The many potentially cytotoxic compounds in cigarette smoke (e.g., carbon monoxide, aldehydes, ketones, nitrosamines, dihydroxybenzenes) [119] may directly compromise neuronal and cellular membrane function of cerebral tissue. There are several potential mechanisms that may contribute independently, or in concert, to the neurobiological and neurocognitive abnormalities in chronic smokers. These mechanisms may operate in a direct and/or indirect manner. The following overview is based on *in vivo* and *in vitro* studies of animals and humans.

### 5.1. Direct Mechanisms

A significant number of potentially cytotoxic compounds (e.g., carbon monoxide, free radicals and their precursors, nitrosamines, phenolic compounds, and other polynuclear aromatic compounds [119]), are found in the gas and particulate phases of cigarette smoke, which may be directly cytotoxic, damage neuronal or glial cell organelles and promote oxidative damage ([120], Muscat, 2004 #13479, [121,122]). For example, carbon monoxide (CO) levels are significantly higher in smokers [123], and this elevation is associated with decreased effective hemoglobin concentrations, diminished oxygen carrying capacity of erythrocytes [124], as well as a diminished efficiency of the mitochondrial respiratory chain [125]. Furthermore, cigarette smoke also contains high concentrations of free radical species (e.g., reactive nitrogen species; reactive oxygen species, ROS) known to promote oxidative damage or stress to cellular structures as well as to macromolecules including membrane lipids, proteins, carbohydrates and DNA [126]. The radical species in the particulate matter of cigarette smoke are long-lived (*i.e.*, hours to months) compared to those in the gas phase [5], and can compromise organs other than the lungs [120,127]. *In vivo* chronic exposure of rat brain tissue to cigarette smoke significantly decreases membrane-bound ATPases, which alters ion homeostasis, and leads to increased Ca^2+^ and Na^+^ levels in the cytosol of various cell types [128], as well as increased Ca^2+^ in mitochondria [122], which is associated with neuronal injury or death [129]. Increased mitochondrial Ca^2+^ secondary to cigarette condensate exposure is associated with damage to the inner mitochondrial membrane (e.g., membrane swelling) and vacuolization of the matrix. Importantly, nicotine delivered independently of cigarette smoke does not appear to produce these adverse affects [122]. Nicotine administration in adolescent rats does, however, evoke cell injury and loss throughout the brain, with significant effects in the hippocampus of female rats but not males [130,131]. In general, the mechanisms underlying the observed nicotine-induced cell injury remain to be fully explicated.

### 5.2. Indirect Mechanisms

*In vivo* chronic cigarette smoke exposure is also associated with decreased enzyme-based free radical scavenger (e.g., superoxide dismutase, catalase, glutathione reductase) and non-enzyme-based radical scavenger (e.g., glutathione and vitamins A, C and E) concentrations in rat brains [132,133]. This may render brain tissue more vulnerable to oxidative damage by radical species generated by cellular metabolism or other exogenous sources. The brain, in general, is exceedingly susceptible to oxidative damage because of high levels of unsaturated fatty acids in the composition of cell membranes and myelin. Additionally, chronic cigarette smoking is related to nocturnal hypoxia [7] as well as chronic obstructive pulmonary disease and other conditions that may impair lung function [8]. Decreased lung function is associated with poorer neurocognition and increased subcortical atrophy in older adults [134]. Chronic smoking increases the risk for atherosclerosis [9], as well as abnormalities in vascular endothelial morphology and function [135–138], which may alter cerebral perfusion. Additionally, nicotine administered through means other than cigarette smoke may alter or impair vasomotor reactivity of cerebral arterioles through upregulation of Ca^2+^ channels and/or modulation of nitric oxide [136]. These processes may impact the functional integrity (e.g., vasomotor reactivity/responsivity) of the cerebrovasculature and may, at least partially, contribute to the decreased regional cerebral blood flow [114,115,139] and/or white matter disease [85,87–89,140,141] observed in chronic smoking. Both the neocortex and underlying WM are vulnerable to the effects of diffuse ischemia (see [142] and references therein). Correspondingly, it has been suggested that late-myelinating areas such as the frontal and temporal lobes may be particularly vulnerable to increased oxidative stress and cerebral hypoperfusion [143,144], both of which have been described in chronic smokers.

Chronic smoking is also associated with central obesity (often reflected in increased body mass index; BMI) and/or insulin resistance [145], which, in turn, are reported to adversely affect brain neurobiology [146–149] and neurocognition [146,150].

In summary, although nicotine is likely the principal bioactive agent that underlies the addictive properties of tobacco smoke [19,151–154], the reviewed literature suggests that the majority of adverse neurobiological and neurocognitive effects of chronic cigarette smoking are a function of the direct and indirect consequences of continual exposure of the cardiopulmonary system, cerebrovascular system and brain parenchyma to the combination of non-nicotine combustion products contained in cigarette smoke [13,14,155]. However, a significant amount of data regarding potential mechanisms contributing to the neurobiological and neurocognitive abnormalities observed in humans is derived from *in vitro* and animal studies. Consequently, it is unclear if all potential mechanisms are generalizable to humans.

## 6. Discussion

The cumulative body of research reviewed suggests chronic cigarette smoking is associated with deficiencies in auditory-verbal learning and/or memory, general intellectual abilities, visual search speeds, processing speed, cognitive flexibility, working memory and executive functions, across a wide age range. With advancing age, chronic smoking is related to abnormal decline in reasoning, memory and global cognitive function, and may increase the risk for both vascular dementia and Alzheimer’s Disease. However, several studies showed a weak or no association with smoking status and neurocognition. Chronic smoking is related to structural and biochemical abnormalities in multiple brain regions, particularly the anterior dorsolateral, mesial frontal cortex, limbic system and underlying WM. A dose-response relationship is suggested between cigarette smoking, neurocognition and neurobiological function. The reviewed literature suggests the adverse neurobiological and neurocognitive effects of chronic cigarette smoking in humans may be related to the direct and indirect consequences of continual exposure of the cardiopulmonary system, cerebrovascular system and/or brain parenchyma to the combustion products of cigarette smoke. However, the potential mechanisms contributing to the neurobiological abnormalities observed are derived from *in vitro* and animal studies. Consequently, it is unclear if these mechanisms are actually operational in humans. Furthermore, it is uncertain to what extent, if any, the reported neurocognitive and neurobiological abnormalities reported in smokers are influenced by premorbid or comorbid factors. Overall, the following methodological limitations are present in the reviewed literature:

### 6.1. Confounding Variables

Potentially confounding medical conditions (e.g., hypertension, diabetes, insulin-resistance, chronic obstructive pulmonary disease, atherosclerosis, neurodegenerative diseases) and comorbid alcohol use/misuse, substance use/misuse, and psychiatric conditions (particularly mood disorders) were not consistently screened or statistically accounted for in many studies. Several psychiatric disorders known to have adverse effects on brain neurobiology and neurocognition are highly prevalent in chronic smokers, including anxiety disorders [156], attention deficit/hyperactivity disorder [157,158], alcohol and substance use disorders [13,157,159], mood disorders [160,161], and schizophrenia-spectrum disorders [162,163]. Additionally, the potential influence of sex, exercise, diet, body mass index, exposure to secondary/environmental smoke, nicotine withdrawal and genetic predispositions [e.g., ApoE4 genotype, single nucleotide polymorphisms in nicotinic acetlycholinergic receptors (nAChr), brain derived neurotrophic factor (BDNF), dopamine receptor D2 (DRD2), catechol-O-methyl transferace (COMT)] were not considered. The aforementioned factors are likely mediators or moderators of brain neurobiology and neurocognition in controls and addictive disorders [146,147,149,164–181]. Finally, the potential effects of nicotine withdrawal on the primary measures of interest were not addressed in many studies.

### 6.2. Limited Scope of Neurocognitive Assessment

Overall, there are a limited number of studies in each age group that conducted a comprehensive assessment of neurocognition. Additionally, measures of executive function (e.g., Categories Test, Wisconsin Card Sorting Test, Wechsler Adult Intelligence Scale-III Matrix Reasoning) were seldom administered. In older adults, many of the population-based research used single screening measures of global cognitive function (e.g., MMSE), or employed a composite score based on a limited number of tests primarily used to assess the severity of cognitive dysfunction in neurodegenerative diseases. Additionally, only two studies [29,63] investigated the effects of chronic smoking on tasks specifically assessing decision making, risk taking and impulsivity. Consequently, the full scope of the neurocognitive consequences associated with chronic smoking remains unclear.

### 6.3. Limited Number of Neurocognitive Studies in Young Adults

The vast majority of studies investigating the neurocognitive consequences of chronic cigarette smoking have been conducted in middle aged and older adults. There is a particular shortage of studies in the 30–40 years of age range.

### 6.4. Limited Number of Neuroimaging Studies

Previous neuroimaging research assessing the *chronic* effects of cigarette smoking has been primarily restricted to a few CT and MR-based studies of brain morphology, metabolites or blood flow, which primarily targeted neocortical and subcortical GM. Only one study investigated WM integrity via DTI. Prospective multimodal neuroimaging studies thoroughly examining WM morphology, biochemistry and perfusion of regional cerebral WM have not been conducted. Assessment of the cerebral WM is vital to better understand the extent of potential neurobiological dysfunction associated with chronic cigarette smoking.

### 6.5. Limited Longitudinal Research

The vast majority of studies assessing the neurocognitive and neurobiological consequences of chronic smoking are cross-sectional in design. The few longitudinal neurocognitive and neuroimaging-based studies were conducted with older adult cohorts.

### 6.6. Absence of MR-based Studies Examining Relationships between Brain Neurobiology and Neurocognition

No study has concurrently combined MR-based neurobiological measures with comprehensive neurocognitive assessment in order to study the correspondence between brain function and neurocognition. Studies relating MR-based brain volumetric and metabolite measures to neurocognition in *substance dependent* populations have observed different patterns/relationships for smokers and non-smokers [182,183] suggesting a differential use of compensatory functions in smokers and non-smokers to accomplish the same task.

## 7. Conclusions

Increasing evidence suggests that chronic smoking in community-dwelling participants is associated with diminished function of multiple neurocognitive abilities and neurobiological abnormalities. The cumulative pattern of neurocognitive findings suggests dysfunction prominently in neurocircuitry implicated in decision making, impulse control, judgment, planning and reasoning skills, and in the initiation and maintenance of substance use disorders [184–187]. Specifically, the pattern of the neurocognitive and neurobiological findings in chronic smokers points to abnormalities in the brain reward system [186–188]. Major components of the brain reward system include (but are not limited to) the dorsolateral prefrontal cortex, orbitofrontal cortex, insula, anterior cingulate cortex, hippocampus, amygdala, nucleus accumbens, ventral tegmental area and other nuclei in the basal forebrain and ventral pallidum [186,189–191]. Plastic changes in the brain reward system are implicated in the development and maintenance of all substance use disorders, including nicotine dependence, and other maladaptive behaviors [186–188,192–194]. However, the actual mechanisms promoting the neurocognitive and neurobiological abnormalities reported in chronic smokers are unclear and premorbid variables(e.g., genetic vulnerabilities) must also be considered as potential contributing factor. More specifically, the neurobiological and neurocognitive abnormalities reported in the reviewed studies may represent premorbid risk factors for the development and maintenance of nicotine dependence and/or premorbid vulnerabilities that were compounded by the effects of chronic smoking. Additionally, as many studies of the neurocognitive consequences of chronic smoking were conducted with older adults, the reported findings may be influenced by a survivor effect [43].

To assist in clarifying the factors contributing to the reported neurocognitive and neurobiological dysfunction, studies are needed that:

1. Concurrently assess cohorts of males and females ranging from young to older adults.
2. Employ prospective multi-modality neuroimaging studies (*i.e.*, combining brain morphology, biochemistry, perfusion, and metabolism in the same cohort), with particular attention to the brain reward system.
3. Employ comprehensive neurocognitive testing including behavioral measures of impulsivity, decision-making and risk taking [24,195,196].
4. Consider genetic factors (e.g., ApoE genotype, single nucleotide polymorphisms in BDNF, nAChr, DRD2, COMT, glutamate receptors) implicated in the development and maintenance of substance use disorders (see [197–200]). Such an approach would better delineate the extent and magnitude of the neurobiological and neurocognitive consequences of chronic cigarette smoking, the roles of common genetic variations in vulnerability to nicotine dependence and their inter-relationships.
5. Employ prospective serial longitudinal studies to assess changes in neurobiology and neurocognition over extended periods in chronic smokers (e.g., >5 years). Additionally, it is vital to conduct prospective pre-and-post neuroimaging and neurocognitive studies with individuals engaging in smoking cessation programs to determine if smoking-related neurobiological and neurocognitive abnormalities recover with smoking cessation, and to assess the effect of pharmacologic interventions (e.g., nicotine replacement, varenicline) on neurobiological and neurocognitive changes. Such longitudinal studies will assist in determining if the neurocognitive and/or neurobiological abnormalities observed in cross-sectional studies are related to premorbid factors.

In conclusion, chronic cigarette smoking appears to be associated with demonstrable abnormalities in brain neurobiology and neurocognition in cross-sectional research across the lifespan, and is related to abnormal rates of brain volume loss in the elderly. However, the mechanisms promoting these abnormalities have yet to be explicated in humans. To better understand the factors associated with the reported neurocognitive and neurobiological abnormalities, longitudinal research combining comprehensive neurocognitive assessment with neuroimaging of brain metabolites, microstructure, macroscopic morphology, brain function and genetic vulnerabilities are necessary. Such longitudinal studies are required to inform the development of more effective pharmacological and behavioral interventions to reduce the ever-increasing worldwide mortality and morbidity associated with the modifiable health risk that is chronic cigarette smoking.

## Figures and Tables

**Table 1.** Neurocognitive studies of chronic smoking in adults (sorted by age group, then year of publication).

**Authors [reference number]**	**Smokers (n)**	**NSC (n)**	**Age group/Mean age or (range)**	**Neurocognitive measures or domains assessed**	**Major findings** (all reported findings are statistically significant unless otherwise indicated)
Jacobsen *et al.* (2005) [23]	41 current	32	Adolescents & Young adults/16.8 ± 1.2	Hopkins Verbal Learning Test-Revised n-back task (measure of working memory, Connors Continuous Performance Task, auditory and visual selective attention, verbal and visuospatial divided attention	Smokers demonstrated poorer working memory than NSC. Earlier age of smoking onset was related to poorer working memory. Male smokers were inferior to female smokers on measures of selective and divided attention.
Spilich *et al.* (1992) [71]	45 current	45	Young adults/19.2 ± 1.2	Visual search speed/accuracy, sustained visual attention, working, memory, information processing speed	Smokers performed worse than NSC on all measures of sustained attention and information processing speed.
Elwan *et al.* (1997) [70]	60 current	69	Young adults through older adults/49.9 ± 3.8 (20–76)	Paced Auditory Serial Attention Test, Trail Making Test A and B	No significant differences observed between smokers and NSC on any measure.
Lejuez *et al.* (2002) [72]	26 current	34	Young adults/20.1 ± 2.8	Balloon Analogue Risk Task (BART), Iowa Gambling Task (IGT)	Smokers demonstrated increased risk-taking levels on the BART compared to NSC. Smokers and NSC showed no differences on the IGT.
Fried *et al.* (2006) [25]	27 current 11 former	64	Young adults/(17–21)	WAIS-III, Wechsler Memory Scale-III, Peabody Picture Vocabulary, Test of Variables of Attention	Overall, current smokers performed worse than NSC on measures of receptive and expressive language, oral arithmetic and auditory-verbal memory.
Yakir *et al.* (2007) [24]	91 current 40 former	151	Young adults (all female)/23.9 ± 2.2	CogScan (v4.0): a comprehensive battery assessing information processing speed, sustained attention, fine motor skills, auditory-verbal and visuospatial memory, reasoning and impulsivity.	Current smokers showed poorer sustained attention, impulse control and planning/reasoning than NSC. Former smokers had poorer impulse control and planning/reasoning than NSC. Current and former smokers were not significantly different on any measure.
Weiser *et al.* (2009) [27]	5762 current 695 former	13,764	Young adults (all males)/(18–21)	Measures of verbal comprehension, verbal and non-verbal abstraction, and mathematical knowledge, Individual measures combined to form composite score of general IQ.	Current and former smokers performed worse than NSC, although the difference between former smokers and NSC was trivial with respect to effect size after adjustment for socioeconomic status. Current smokers who smoked more than 11 cigarettes per day showed the poorest performance relative to NSC.
Ernst *et al.* (2001) [35]	14 current 15 former	9	Young and Middle aged adults/(21–45)	Domains assessed were verbal reasoning and working memory.	Current smokers and former smokers showed poorer working memory than NSC. Current smokers had poorer working memory than former smokers.
Sakurai and Kanazawa (2002) [62]	20 current	20	Young and Middle aged Adults/(23–41)	Measures of auditory-verbal learning and memory, mental arithmetic, and verbal fluency	No differences between smokers and NSC on any task.
Paul *et al.* (2006) [33]	62 current	62	Young and middle aged adults/28.1 ± 7.2, 55.2 ± 7.3	Domains assessed included executive function, finger tapping speed, learning and memory, sustained attention, word fluency, working memory	Smokers performed more poorly than NSC on one measure of executive function. Middle aged smokers showed poorer auditory-verbal memory than aged NSC and young adult smokers.
George *et al.* (2002) [36]	29 current	16	Middle aged adults/41.5 ± 10.3	Domains assessed were visuospatial working memory, cognitive flexibility	Smokers exhibited worse visuospatial working memory.
Schinka *et al.* (2002) [67]	174 current 80 former	204	Middle aged adults/38.4 ± 2.3	CVLT, WAIS-R Block Design, Rey-Osterreith Complex Figure, Wisconsin Card Sorting Test, Paced Auditory Serial Attention Test, Grooved Pegboard, semantic fluency, global cognitive function	Higher pack years of smoking was related to lower global cognitive functioning.
Kalmijn *et al.* (2002) [32]	529 current 715 former	619	Middle aged adults/56.4 ± 7.1	Domains assessed were auditory-verbal and visuospatial learning and memory, cognitive flexibility, processing speed, global cognitive function	Current smokers showed poorer cognitive flexibility and processing speed than NSC. No differences between former smokers and NSC on any measure.
Sabia *et al.* (2008) [43]	815 current 2,030 former	2,543	Middle age adults/56 ± 6	Mill Hill Vocabulary Test, measures of verbal and semantic fluency, verbal and mathematical reasoning, auditory verbal learning	In cross-sectional analyses, smoking history was associated with increased risk of poor memory. Over 4–7 years, current smokers and recent former smokers showed significantly greater declines in reasoning than never smokers. No significant declines in cognitive function were observed in former smokers.
Cerhan *et al.* (1998) [56]	13,913 total participants, numbers of current, former smokers and NSC not provided	NA	Middle age and older adults/(45–69)	WAIS-R Digit Symbol Test, measures of auditory-verbal memory and verbal fluency	Current smokers demonstrated poorer performance on Digit Symbol and auditory-verbal memory. For smokers, greater lifetime number of cigarettes was related to poorer Digit Symbol and auditory-verbal memory performance.
Hill *et al.* (2003) [30]	164 current	438	Middle aged & older adults/NA	WAIS-R Block Design and measures of auditory-verbal learning and memory, general knowledge, word comprehension	Smokers, irrespective of age performed worse than NSC on Block Design and on measures of auditory-verbal memory.
Razani *et al.* (2004) [73]	125 former or never smokers. Groups were retro-spectively divided into non/light, moderate heavy and heavy smokers based on pack years. Only 2 of 127 subjects were active smokers.	NA	Middle aged & older adults 65.9 ± 8.3	WAIS-R Digit Symbol and Digit Span, WMS-R Logical Memory and Visual Reproduction, Rey-Osterrieth Complex Figure—Immediate Recall, Stroop Word and color trials, WCST	Heavy smokers performed worse than moderate smokers and non/light smokers on the WCST.
Hill (1989) [42]	11 current 12 former	53	Older adults/71.6 ± 4.9	WAIS-R Block Design, Digit Symbol, and Digit Span; WMS Logical Memory and Associative Memory, Bender Gestalt, Cross Off Task, word fluency and Digit Symbol.	At the baseline assessment current smokers performed worse than former smokers and NSC on the Cross Off task. At reassessment (15 months after baseline), current smokers performed worse than former and current smokers on the Cross Off Task and Digit Symbol.
Hebert *et al.* (1993) [69]	current former		Older adults/(65 to ≥ 80)	Measures of auditory-verbal memory, working memory and orientation	Current and former smokers showed no significant decline over a 3-year period on any measure relative to NSC after control for age, sex, education and income.
Launer *et al.* (1996) [53]	110 current 288 former	91	Older adults/75 ± 4.5	MMSE	Smokers performed worse on the MMSE than NSC after correction for age, education and alcohol consumption. Over a 3-year period, current smokers with cardiovascular disease and/or diabetes showed the greatest decline in MMSE scores.
Ford *et al.* (1996) [68]	259 current and former smokers combined	369	Older adults/>75	Pfeiffer Short Portable Mental Status Questionnaire (PSPMSQ)	Baseline and change over 4 years on the PSPMSQ was not associated with smoking status.
Galanis *et al.* (1997) [46]	921 current 1,334 former	1,174	Older adults/77.4 ± 4.6	Cognitive Abilities Screening Test (CASI): includes task of attention, concentration abstraction, judgment, verbal and verbal fluency. A composite CASI score was formed from the individual components.	After adjustment for age, education and Japanese acculturation, current and former smokers had lower CASI score than never smokers. Higher risk of impaired performance on CASI scores was associated with current and former smoking.
Edelstein *et al.* (1998) [34]	114 current	407	Older adults/72.0 ± 9.2	MMSE, Trail Making Test Part–B, Buschke Selective Reminding Test, Modified Version of WMS Visual Reproduction, semantic fluency and auditory-verbal memory.	No differences between male smokers and male NSC. Female smokers demonstrated poorer performance than female NSC on the MMSE.
Cervilla *et al.* (2000) [51]	80 current 204 former	134	Older adults/(65–95)	Organic Brain Syndrome Scale (OBS) (measure of orientation to person time, place and context)	After controlling for sex, age, alcohol consumption, education, depression and baseline cognitive function, current smokers had a 3.7 fold risk of impaired performance on the OBS after one year.
Schinka *et al.* (2002) [64]	334 participants with various smoking and alcohol use histories.	61	Older adults/(60–84)	MMSE, Hopkins Verbal Learning Test, Stroop Color Word test, Trail Making Test Part–B	No significant effects were found for alcohol or cigarette consumption on any measure.
Chen *et al.* (2003) [66]	195 current 51 former	68	Older adults/72 ± 6	Cognitive Abilities Screening Instrument (measure of global cognitive function)	No significant group differences on the Cognitive Abilities Screening Instrument.
Deary *et al.* (2003) [39]	126 current 278 former	387	Older adults/75.6 ± 5.4	Moray House Test (MHT). Included measures of verbal, numerical, and verbal reasoning. Global MHT score formed from the individual components.	After adjusting for MHT score at age 11 years of age, education and sex, current smokers had lower MHT scores than NSC and former smokers. NSC and former smokers were not different.
Schinka *et al.* (2003) [65]	30 current	86	Older adults/(60–84)	MMSE, Hopkins Verbal Learning Test, Stroop Color-Word Test	Pack years significantly predicted MMSE and auditory-verbal memory scores, but only accounted for 1.8% of variance in auditory-verbal memory.
Huadong *et al.* (2003) [44]	720 current 276 former	1,976	Older adults/>60	MMSE	Current smokers had 2.3 greater risk of impaired MMSE score (*i.e.*, <17) relative to NSC after adjustment for age, sex, education, occupation and alcohol use.
Ott *et al.* (2004) [50]	2,037 current 3,372 former	3,800	Older adults/>65	MMSE	Current smokers relative to never smokers showed a greater rate of decrease in MMSE scores over approximately 2 years controlled for age, sex, education, baseline MMSE, history of myocardial infarction, and cerebrovascular accident. Higher pack years was associated with higher rate of decline in MMSE.
Reitz *et al.* (2005) [54]	90 current 135 former	184	Older adults/75.6 ± 5.4	MMSE, Boston Diagnostic Aphasia Evaluation: Boston Naming Test, Category Naming, Phrase Repetition, Complex Ideational Material, WAIS-R Similarities, Nonverbal Identities and Oddities from The DRS, Rosen Drawing Test, Buschke Selective Reminding Test, Benton Visual Retention Test.	Over approximately five years, there was no association between current or former smoking status and change in cognition in those <75 years of age. For those >75 years of age, current smokers showed greater decline in memory than former smokers and NSC. The memory declines were greatest in current smokers who were *not* carriers of the ApoEɛ4 allele.
Whalley *et al.* (2005) [41]	90 current 135 former	184	Older adults/64	Raven’s Standard Progressive Matrices, Rey Auditory Verbal Learning Test, WAIS-R Digit Symbol and Block Design, Uses of Common Objects Test and a composite measure of all tests.	After adjusting for childhood IQ, age, education, occupation, lung function, any history of smoking was associated with lower scores on Digit Symbol.
Fischer *et al.* (2006) [47]	262 current 75 former	NA	Older adults/75	MMSE	Longer duration of smoking was associated with lower MMSE after adjusting for vascular risk factors and use of antihypertensive medication.
Stewart *et al.* (2006) [45]	135 current 246 former	217	Older adults/64.5 ± 6.5	Raven’s Standard Progressive Matrices, Rey Auditory Verbal Learning Test, Trail Making Test, Digit Symbol Test, Mill Hill Vocabulary Scale (MHS), MMSE and a composite measure of all tests.	In men, after adjusting for age, blood pressure and total cholesterol, higher pack years was associated lower scores on Auditory-verbal leaning, Digit Symbol Test, MHS in men. In women, higher pack years was associated with lower MMSE. In women, current smoking status was associated with poorer auditory-verbal learning.
Starr *et al.* (2006) [31]	289 total participants. Number of smokers, NSC, not provided	NA	Older adults/64 and 66	Raven’s Standard Progressive Matrices, Rey Auditory Verbal Learning Test, WAIS-R Digit Symbol and Block Design, Uses of Common Objects Test	Current smokers performed worse NSC and former smokers on auditory-verbal learning and information processing speed after adjusting for childhood IQ.

Note. DRS: Mattis Dementia Rating Scale; MMSE: Mini Mental Status Examination; NA: Not available; NSC: non-smoking (never-smoker) control; WAIS-III: Wechsler Adult Intelligence Scale-3rd Edition; WAIS-R: Wechsler Adult Intelligence-Revised; WMS-R: Wechsler Memory Scale-Revised.

**Table 2.** Computerized tomography and magnetic resonance neuroimaging studies of chronic smoking in adults (sorted by imaging modality, then age group).

**Authors [reference number]**	**Smokers (n)**	**NSC (n)**	**Age group/mean age or (range)**	**Neuroimaging modality**	**Major findings** (all findings are statistically significant unless otherwise indicated)
Akiyama *et al.* (1997) [74]	104 current and former smokers combined	173	Young to older adults/(22–89)	CT (volumetric and cortical perfusion)	A history of smoking (*i.e.*, current or former smoking) was associated with lower global cerebral perfusion after control for hypertension, WM disease and age. Over approximately 3 years, smokers showed greater decrease in global perfusion and greater global atrophy than NSC.
Kubota *et al.* (1987) [76]	159 current Non-smoking group contained 177 never and 17 “light” smokers	NA	Middle aged and older adults/(40–69)	CT	Current smokers from 50 to 69 showed greater global atrophy than never/light smokers.
Hayee *et al.* (2003) [75]	219 current, Non-smoking group contained 183 never smokers and 17 “light” smokers	NA	Middle aged and older adults/(40–70)	CT	Current smokers between 50–70 years of age showed greater global atrophy than the non-smoking group.
Brody *et al.* (2004) [79]	19 current	17	Young to older adults/(21–65)	MRI	Smokers showed lower volumes and densities in the anterior frontal lobe GM, smaller volume of the left dorsal anterior cingulate gyrus, and lower GM density of the right cerebellum relative to NSC. Higher pack years was associated with lower anterior frontal GM.
Gallinat *et al.* (2006) [80]	22 current	23	Adults/30.6 ± 7.7	MRI	Smokers demonstrated smaller GM volumes and densities in frontal, temporal and occipital regions compared to NSC. Smokers also showed lower volume or density in the thalamus, cerebellum and other subcortical nuclei/regions. Higher pack years was related to lower frontal, temporal and cerebellar GM volume.
Paul *et al.* (2008) [82]	10 current	10	Middle aged adults/38.5 ± 13.4	MRI (diffusion tensor imaging)	Smokers showed higher fractional anisotropy (FA) in the body and whole corpus callosum than NSC. Smokers with low Fagerstrom Test for Nicotine Dependence scores (mean = 1.6) showed higher FA in the whole corpus callosum than smokers with high scores (mean = 5.6).
Longstreth *et al.* (2000) [78]	3,301 total participants, numbers of NSC, and smokers not provided	NA	Older adults/>65	MRI	Over 5 years, higher pack years in men was related to increased ventricular volume in men and associated with increased sulcal volume in women, after control for age and vascular risk factors.
Almeida *et al.* (2008) [81]	39 current	39	Older adults/75.4 ± 3.3	MRI	Smokers showed decreased GM densities in the posterior cingulate gyrus and precuneus bilaterally, right thalamus and right precentral gyrus.
Epperson *et al.* (2005) [102]	16 current	20	Adults/34 ± 11	MRS	Gamma-aminobutyric acid (GABA) levels n the occipital GM were not different between NSC and male smokers. Female smokers showed a significant reduction in GABA levels during the follicular phase of the menstrual cycle. GABA levels showed no changes after 48 hours of smoking cessation in both males and females.
Gallinat *et al.* (2007) [97]	13 current	13	Adults/36.6 ± 10.1	MRS	Smokers showed lower N-acetylaspartate levels than NSC in the left hippocampus. Higher pack years was related to higher choline-containing compounds in the anterior cingulate gyrus.
Gallinat and Schubert (2007) [98]	13 current 9 Former	13	Adults/36.1 ± 9.8	MRS	No significant group differences were observed in glutamate levels of the anterior cingulate cortex and left hippocampus.

Note. CT: computed tomography; GM: Gray Matter; MRI: magnetic resonance imaging; MRS: magnetic resonance spectroscopy; NA: not available; NSC: non-smoking (never-smoker) control; WM: white matter.
